# Evaluation of professional nursing practice environments in primary health care

**DOI:** 10.3389/fpubh.2024.1477067

**Published:** 2025-01-22

**Authors:** Letícia de Lima Trindade, Bruna de Campos Guerreiro, Sandra Mara Soares de Oliveira, João Miguel Almeida Ventura-Silva, Leticia Rostirolla, Samuel Spiegelberg Zuge, Olga Maria Pimenta Lopes Ribeiro

**Affiliations:** ^1^Universidade Comunitária da Região de Chapecó, Programa de Pós-graduação em Ciências da Saúde, Chapecó, Santa Catarina, Brazil; ^2^Universidade Comunitária da Região de Chapecó, Departamento de Enfermagem, Chapecó, Santa Catarina, Brazil; ^3^Escola Superior de Saúde Norte da Cruz Vermelha Portuguesa, Oliveira de Azeméis, Portugal; ^4^Escola Superior de Enfermagem do Porto, Cintesis@RISE, Porto, Portugal

**Keywords:** nursing, quality of health care, work environment, professional practice, evaluation of patient care outcomes

## Abstract

**Objective:**

Analyzing the environments of professional nursing practice in Primary Health Care in a municipality in the west of the state of Santa Catarina.

**Method:**

A quantitative, explanatory, cross-sectional study was carried out in 24 Primary Care health units. A total of 159 nursing professionals took part and answered a questionnaire on personal and professional characteristics and the Nursing Professional Practice Evaluation Scale/SEE-Nursing Practice, comprised of Structure, Process, and Outcome sub-scales. The data was subjected to descriptive and inferential analysis.

**Results:**

There were positive evaluations for people management and leadership in the Structure dimension. Technicians and nursing assistants had positive evaluations of the organization and sustainability of practice. In Process, collaboration and teamwork stood out, and strategies to guarantee the quality of care. In the Outcome dimension, there was a relationship between gender and systematic evaluation of care, with higher scores among women.

**Conclusion:**

A positive evaluation of professional nursing practice environments in Primary Care contributes to the effective management of people, materials, and care processes, reflecting on the quality of nursing practice and access to healthcare.

## Introduction

Primary Health Care (PHC), known in Brazil as Basic Care (BC), is an essential strategic point for achieving the fundamental principles of the Unified Health System (*Sistema Único de Saúde*, SUS), such as universality, integrality, and equity. Through a broad spectrum of activities – including promotion, prevention, protection, diagnosis, treatment, rehabilitation, harm reduction, palliative care, and health surveillance – PHC represents the first level of contact between individuals and the health system, promoting continuous and comprehensive health care. This approach reinforces the commitment to public health, aligning with the needs of the population and contributing to the sustainability of the health system (1–3).

In the Brazilian context, the Ministry of Health prioritizes the organization of PHC within the SUS as one of its main objectives, developing the Family Health Strategy (FHS) to qualify, expand, and consolidate this first level of care. The FHS facilitates the restructuring of health work processes, promoting more accessible and personalized care, which is crucial for the effectiveness of the health system (1, 4).

Family health teams are made up of general practitioners, nurses, nursing technicians, community health agents, and some teams include oral health professionals. The teams work in Basic Health Units, operating 40 h/week, serving a registered population of 2,000 to 3,500 people, located within their territory. The teams develop local action plans, focusing on preventive interventions and health promotion, in addition to meeting spontaneous demands. Home visits are essential, allowing continuous monitoring of chronic patients, pregnant women, children, and other vulnerable populations.

In PHC, teamwork is fundamental to meeting user demands. The Family Health teams (FHt) are multidisciplinary, made up of general practitioners, nursing technicians or assistants, nurses, and community health agents, with a significant role for nursing professionals in absorbing these demands. For effective care, it is imperative that the team acts in an integrated and multi-professional manner, creating a professional practice environment that favors the quality of the care provided (5).

The quality and configuration of professional practice environments are fundamental not only for fostering positive interpersonal relationships and professional autonomy, but also for effective management of their structural aspects. These environments directly influence the satisfaction and performance of healthcare teams and contribute to user safety. These elements are crucial to the development of efficient and safe healthcare practices, highlighting the importance of well-managed environments in the context of healthcare (6, 7).

The evaluation of these environments can be structured through Donabedian’s triad – Structure, Process, and Outcome – which serves as a theoretical model for the analysis and continuous improvement of the quality of health services. Structure involves the quality of the physical and organizational properties of the environment in which care takes place, while the Process defines the nursing interventions carried out in the user’s care, finally, Outcome measures the end of this analysis, that is, whether the structure and processes made a difference to the quality of the work (8).

This theoretical framework has been widely explored in studies of public health contexts, to evaluate nursing environments favorable to quality and care (9). Thus, the following research question emerged: how are professional health practice environments characterized in PHC in Santa Catarina, Southern Brazil? The aim of the study is to evaluate the professional nursing practice environment in Primary Health Care in a municipality in the west of Santa Catarina, southern Brazil.

## Materials and methods

This is a quantitative, exploratory, cross-sectional study, anchored in the theoretical framework known as the Donabedian Triad (8).

The research was carried out in 24 basic health units (BHUs) in a municipality in the west of Santa Catarina, which has a total of 25 BHUs and one prison BHU, in which 62 FHS work with a maximum of 110 teams. The scenario has a population coverage of PHC of 100% and FHS of 89.2%3. The FHS is made up of doctors, nurses, nursing assistants and technicians, community health agents, endemic disease agents, and if expanded, also has an Oral Health team (OHt), each team being responsible for the health of a given territory.

Although the study was not conducted in prison units, it reached teams that work in different contexts, including urban and rural environments, in more and less vulnerable contexts. All units were included in the study, and all eligible professionals, according to the criteria, were approached personally in the units, with all professional categories represented (mid-level and higher-level professionals). The sample is culturally representative, especially in the Brazilian context, since it considers the composition of the nursing team in the PHC care model in Brazil.

The selection criteria for the participants were: working as a nurse, nursing technician, or nursing assistant at the FHS, with a period of experience in the service equal to or greater than 3 months. Professionals on leave for any reason during the data collection period were excluded.

The sample was defined based on a population of 238 professionals available in the scenarios, assuming a margin of error of 5% and a 95% confidence interval, thus the survey sample was 147 participants, with 159 participants selected for convenience. To achieve this goal, all the professionals who met the criteria were invited to take part in the study. Of these, 159 agreed to take part, 44 were nurses, 23 were nursing technicians and 92 were nursing assistants. During the data collection process, the prison BHU was excluded due to the security rules and specificities of the context, and in one BHU there were no participants who met the inclusion criteria.

Data collection took place in person between July and September 2022. The nursing professionals received sealed envelopes containing a presentation of the study, the Free and Informed Consent Form and the data collection instruments, that is, the personal and professional data questionnaire, the EAAPPE/ SEE-Nursing Practice Scale, as well as instructions for the envelopes to be re-sealed after completion and delivered to the BHU manager. After the envelopes had been filled in, the researchers returned to the unit to collect them, by prior appointment with the services and the professionals.

For data collection, a self-completion questionnaire was used to characterize personal (age, gender, and marital status) and professional (professional training, time working in the profession, time working in the institution and the unit), followed by the Scale for the Evaluation of Professional Nursing Practice Environments (*Escala de Avaliação dos Ambientes de Prática Profissional de Enfermagem*) – EAAPPE/SEE-Nursing Practice (10), validated for Brazil (11), consisting of three sub-scales: SEE-Nursing Practice – Structure, SEE-Nursing Practice – Process and SEE-Nursing Practice – Outcome. The Scale to be used in Brazil was translated into Brazilian Portuguese by two professionals in the language, undergoing only semantic adaptations and cultural terms used in the country.

The SEE-Nursing Practice – Structure, made up of 38 items divided into 6 dimensions: (1) People management and leadership in the service (10 items); (2) Participation and involvement of nurses in the policies, strategies, and functioning of the institution (8 items); (3) conditions for the proper functioning of the service (6 items); (4) organization and Sustainability of nursing practice (6 items); (5) Institutional policy for professional qualification (6 items); (6) Quality and safety of care (4 items).

The SEE-Nursing Practice – Process is made up of 33 items, distributed over 6 dimensions: (1) Collaboration and teamwork (10 items); (2) Strategies for quality assurance in professional practice (9 items); (3) Autonomous practices in professional practice (7 items); (4) Theoretical and legal subsidies for professional practice (4 items); (5) Interdependence in professional practice (3 items).

The SEE-Nursing Practice – Outcome includes 13 items divided into 2 dimensions: (1) Systematic evaluation of nursing care and indicators (7 items) and (2) Systematic evaluation of nurses’ performance and supervision (6 items).

Regarding the final EAAPPE/SEE-Nursing Practice score, the higher the score, the more favorable the professional nursing practice environment is to the quality of care. Regarding the Subscales, the higher the score, the more favorable the structure, process or outcome is considered to be to the quality of care. To analyze the results of the structure, process and outcome components, the instrument establishes the following criteria: score < 35% - component of the professional nursing practice environment that is barely favorable to the quality of care; between 35 and 55% – component of the professional nursing practice environment that is moderately favorable to the quality of care; between 55 and 75% – component of the professional nursing practice environment that is favorable to the quality of care and, finally, >75% – component of the professional nursing practice environment that is very favorable to the quality of care.

To analyze and organize the data, it was entered into a Google Forms form, which was linked to an Excel spreadsheet, and the analyses were carried out using the Statistical Package for the Social Sciences (SPSS), version 28.0.

The quantitative variables were described by mean and standard deviation (±) or median and interquartile range. The categorical variables were described through absolute and relative frequencies. The association between the numerical variables was evaluated using Pearson’s or Spearman’s correlation coefficients. The significance level adopted was 5% (*p* < 0.05).

The Wilcoxon test was used to compare the three components and the total Scale, with a significance level adjusted by the Bonferroni correction of 0.83%. The analysis of the relationship between personal and professional attributes and the components of the SEE-Nursing Practice used the Lilliefors test to test the normality of the variables, the components and the total Scale. To compare means, the Student’s t-test or Analysis of Variance (ANOVA) supplemented by Tukey’s test was used.

In a validation study of the instrument carried out in Portugal10, SEE-Nursing Practice obtained a Cronbach’s alpha of 0.96, SEE-Nursing Practice – Structure a Cronbach’s alpha of 0.95, SEE-Nursing Practice – Process a Cronbach’s alpha of 0.91 and SEE-Nursing Practice – Outcome a Cronbach’s alpha of 0.93. The national validation study11 obtained an internal consistency of 0.956, 0.929 and 0.937 in the SEE - Nursing Practice - Structure, Process and Outcome, while this study obtained 0.95, 0.95 and 0.92 in the same order.

Data collection complied with the ethical precepts regarding research with human beings and the provisions of Resolutions 466/2012 and 510/2016 of the National Health Council, and the research was approved by the CEP under opinion number 5.485.663/2022.

## Results

Table 1 shows the characterization of the participants.

**Table 1:** Characterization of the sample.

Variables	*n* = 159
Age (years old) – Mean ± SD	46.1 ± 18.6
Gender – *n* (%)	
Male	8 (5.0)
Female	151 (95.0)
Marital status (*n* = 154) – *n* (%)	
Without a partner	41 (26.6)
With a partner	113 (73.4)
Schooling level (highest academic degree) – *n* (%)	
High school	71 (44.7)
Undergraduate	42 (26.4)
Specialization	41 (25.8)
Master’s degree	4 (2.5)
PhD	1 (0.6)
Job title – *n* (%)	
Nurse manager	17 (10.7)
Care nurse	27 (17.0)
Nursing technicians	23 (14.5)
Nursing assistant	92 (57.9)
Time in the profession (years) - median (P25 – P75)	12 (7–20)
Time of service in current job (months) - median (P25 – P75)	6 (1.5–9)

SD, Standard Deviation; P25, Percentile 25; P75, Percentile 75.

Database, 2022.

Table 2 shows the findings regarding the evaluation of Structure, Process, and Outcome, based on participants’ assessment of the professional practice environment in the PHC scenarios surveyed.

**Table 2:** Data on the SEE-Nursing Practice, Santa Catarina, 2022.

SEE domains	Mean ± SD	Barely favorable *n* (%)	Moderately favorable *n* (%)	Favorable *n* (%)	Very favorable *n* (%)
Structure	65.2 ± 14.4	4 (2.5)	30 (18.9)	85 (53.5)	40 (25.2)
Factor 1 - People management and leadership in the service	80.3 ± 17.6	4 (2.5)	10 (6.3)	40 (25.2)	105 (66.0)
Factor 2 - Participation and involvement of nurses in the policies, strategies, and functioning of the institution	56.3 ± 20.0	25 (15.7)	47 (29.6)	61 (38.4)	26 (16.4)
Factor 3 - Conditions for the proper functioning of the service	53.8 ± 19.7	26 (16.4)	55 (34.6)	66 (41.5)	12 (7.5)
Factor 4 - Organization and sustainability of nursing practice	66.8 ± 18.1	6 (3.8)	38 (23.9)	67 (42.1)	48 (30.2)
Factor 5 - Institutional policy for professional qualification	65.3 ± 20.7	11 (6.9)	47 (29.6)	52 (32.7)	49 (30.8)
Factor 6 - Quality and safety of nursing care	68.8 ± 19.3	11 (6.9)	23 (14.5)	76 (47.8)	49 (30.8)
Process	76.6 **±** 12.4	0 (0.0)	9 (5.7)	59 (37.1)	91 (57.2)
Factor 1 - Collaboration and teamwork	74.9 ± 13.9	1 (0.6)	9 (5.7)	75 (47.2)	74 (46.5)
Factor 2 - Strategies for quality assurance of care	75.1 ± 15.9	3 (1.9)	13 (8.2)	64 (40.3)	79 (49.7)
Factor 3 - Autonomous practices in professional practice	74.1 ± 14.8	2 (1.3)	12 (7.5)	83 (52.2)	62 (39.0)
Factor 4 - Theoretical and legal subsidies for professional practice	83.3 ± 15.5	1 (0.6)	9 (5.7)	44 (27.7)	105 (66.0)
Factor 5 - Interdependence in professional practice	74.4 ± 18.1	1 (0.6)	28 (17.6)	74 (46.5)	56 (35.2)
Results	65.3 **±** 17.6	9 (5.7)	30 (18.9)	78 (49.1)	42 (26.4)
Factor 1 - Systematic evaluation of nursing care and indicators	69.1 ± 19.0	7 (4.4)	31 (19.5)	65 (40.9)	56 (35.2)
Factor 2 - Systematic evaluation of nurses’ performance and supervision	61.1 ± 19.8	15 (9.4)	50 (31.4)	59 (37.1)	35 (22.0)

*n = 159. Database, 2022.*

Regarding the final score of the EAAPPE/SEE-Nursing Practice Scale, in the Structure domain, People management and leadership in the service, the majority consider the environment to be very favorable to professional nursing practice (66.0%), and in the Process domain, Collaboration and teamwork (57.2%), Strategies for quality assurance of care (49.5%) and Theoretical and legal subsidies for professional practice (66.0%).

Regarding the association between the Scale’s dimensions and factors and the personal and professional variables, there was no statistically significant association (*p* > 0.05), as shown in Table 3.

**Table 3:** Associations between SEE-Nursing Practice and personal and professional variables.

SEE domains	Age	Time in the profession	Time of service in current job
Structure	*r* = 0.047; *p* = 0.556	*r*_s_ = 0.015; *p* = 0.853	***r***_s_ = 0.006; *p* = 0.938
Factor 1 - People management and leadership in the service	*r* = −0.038; *p* = 0.634	*r*_s_ = 0.038; *p* = 0.631	*r*_s_ = −0.047; *p* = 0.558
Factor 2 - Participation and involvement of nurses in the policies, strategies, and functioning of the institution	*r* = 0.059; *p* = 0.460	*r*_s_ = −0.055; *p* = 0.492	*r*_s_ = −0.101; *p* = 0.208
Factor 3 - Conditions for the proper functioning of the service	*r* = 0.065; *p* = 0.414	*r*_s_ = 0.060; *p* = 0.456	*r*_s_ = 0.112; *p* = 0.163
Factor 4 - Organization and sustainability of nursing practice	*r* = 0.087; *p* = 0.277	*r*_s_ = 0.033; *p* = 0.679	*r*_s_ = 0.068; *p* = 0.395
Factor 5 - Institutional policy for professional qualification	*r* = 0.015; *p* = 0.847	*r*_s_ = −0.026; *p* = 0.746	*r*_s_ = −0.014; *p* = 0.864
Factor 6 - Quality and safety of nursing care	*r* = 0.028; *p* = 0.722	*r*_s_ = 0.110; *p* = 0.168	*r*_s_ = 0.132; *p* = 0.098
Process	*r* = −0.056; *p* = 0.486	*r*_s_ = 0.109; *p* = 0.173	*r*_s_ = 0.058; *p* = 0.467
Factor 1 - Collaboration and teamwork	*r* = −0.020; *p* = 0.803	*r*_s_ = 0.045; *p* = 0.575	*r*_s_ = 0.024; *p* = 0.768
Factor 2 - Strategies for quality assurance of care	*r* = 0.012; *p* = 0.880	*r*_s_ = 0.114; *p* = 0.154	*r*_s_ = 0.062; *p* = 0.443
Factor 3 - Autonomous practices in professional practice	*r* = 0.149; *p* = 0.060	*r*_s_ = 0.054; *p* = 0.499	*r*_s_ = −0.123; *p* = 0.124
Factor 4 - Theoretical and legal subsidies for professional practice	-*r* = −0.091; *p* = 0.254	*r*_s_ = 0.114; *p* = 0.151	*r*_s_ = 0.059; *p* = 0.463
Factor 5 - Interdependence in professional practice	*r* = −0.084; *p* = 0.291	*r*_s_ = 0.032; *p* = 0.691	*r*_s_ = 0.054; *p* = 0.503
Results	*r* = −0.044; *p* = 0.581	*r*_s_ = −0.006; *p* = 0.941	*r*_s_ = −0.003; *p* = 0.972
Factor 1 - Systematic evaluation of nursing care and indicators	*r* = −0.068; *p* = 0.396	*r*_s_ = 0.076; *p* = 0.342	*r*_s_ = 0.036; *p* = 0.655
Factor 2 - Systematic evaluation of nurses’ performance and supervision	*r* = −0.013; *p* = 0.869	*r*_s_ = −0.110; *p* = 0.168	*r*_s_ = −0.093; *p* = 0.244

Santa Catarina, 2022.

*r, Pearson’s correlation coefficient; rs, Spearman’s correlation coefficient.*

Gender was significantly associated with the Systematic evaluation of nursing care and indicators, with higher scores among women and a statistically significant difference with factor 1 of the Outcomes domain (*p* = 0.015).

Professionals with graduate degrees (specialization, master’s, or PhD) had significantly lower scores in Strategies for quality assurance of care and Interdependence in professional practice compared to professionals with high school and undergraduate degrees. Professionals with graduate degrees also had significantly lower scores for Processes and Outcomes when compared to those with higher education (Table 4).

**Table 4:** SEE-Nursing Practice associations with training, Santa Catarina, 2022.

SEE domains	High school (*n* = 71)	Undergraduate (*n* = 42)	Graduate studies (*n* = 46)	Value*
Structure	66.1 ± 14.0	67.4 ± 14.5	61.9 ± 14.7	0.163
Factor 1 - People management and leadership in the service	77.8 ± 19.4	82.9 ± 16.2	81.7 ± 15.5	0.263
Factor 2 - Participation and involvement of nurses in the policies, strategies, and functioning of the institution	57.1 ± 20.2	59.5 ± 20.0	52.4 ± 19.5	0.229
Factor 3 - Conditions for the proper functioning of the service	55.6 ± 20.5	55.2 ± 18.8	49.8 ± 19.2	0.259
Factor 4 - Organization and sustainability of nursing practice	68.0 ± 18.5	69.6 ± 17.3	62.3 ± 17.6	0.129
Factor 5 - Institutional policy for professional qualification	65.8 ± 19.1	69.0 ± 21.9	61.1 ± 21.8	0.190
Factor 6 - Quality and safety of nursing care	72.3 ± 17.9	67.7 ± 18.0	64.3 ± 21.6	0.079
Process	77.0 ± 12.8^ab^	80.0 ± 11.6^b^	72.9 ± 11.8^a^	0.024
Factor 1 - Collaboration and teamwork	73.6 ± 13.9	78.4 ± 14.0	73.6 ± 13.6	0.165
Factor 2 - Strategies for quality assurance of care	76.8 ± 16.4^b^	79.3 ± 14.9^b^	68.7 ± 14.5^a^	0.004
Factor 3 - Autonomous practices in professional practice	73.1 ± 16.1	76.1 ± 14.4	73.7 ± 13.1	0.564
Factor 4 - Theoretical and legal subsidies for professional practice	82.2 ± 15.8	86.3 ± 14.6	82.2 ± 15.7	0.335
Factor 5 - Interdependence in professional practice	77.3 ± 17.3^b^	78.2 ± 17.9^b^	66.2 ± 17.4^a^	0.001
Results	66.4 ± 17.3^ab^	69.0 ± 15.2^b^	60.1 ± 19.2^a^	0.047
Factor 1 - Systematic evaluation of nursing care and indicators	70.5 ± 18.4	72.0 ± 18.4	64.1 ± 19.9	0.105
Factor 2 - Systematic evaluation of nurses’ performance and supervision	62.4 ± 18.9	64.0 ± 18.0	56.3 ± 22.1	0.137

* One-way Analysis of Variance (ANOVA); ^a,b^ Equal letters do not differ by Tukey’s test at a 5% significance level.

Nursing technicians and assistants had significantly higher scores in the Organization and sustainability of nursing practice factor, in the Structure domain, and the overall Process scale when compared to nurses (care and managers). Nursing technicians and assistants also scored significantly higher on the Systematic evaluation of nursing care and indicators, and on the global Outcome scale compared to care nurses. In addition, nursing assistants had significantly higher scores in Strategies for quality assurance of care and Interdependence in professional practice compared to nurses (manager and care). In addition, the findings show that nursing assistants also had significantly higher scores than care nurses in relation to the quality and safety of nursing care, of the Structure domain (Table 5).

**Table 5:** Associations between SEE-Nursing Practice data and job title, Santa Catarina, 2022.

SEE domains	Nurse manager (*n* = 17)	Care nurse (*n* = 27)	Nursing Technicians (*n* = 23)	Nursing Assistant (*n* = 92)	Value*
Structure	63.3 ± 10.5	59.3 ± 14.9	67.0 ± 15.1	66.9 ± 14.4	0.088
Factor 1 - People management and leadership in the service	84.6 ± 9.8	82.4 ± 15.1	77.1 ± 23.2	79.7 ± 17.8	0.526
Factor 2 - Participation and involvement of nurses in the policies, strategies, and functioning of the institution	55.3 ± 18.0	47.7 ± 19.8	58.4 ± 19.2	58.6 ± 20.2	0.092
Factor 3 - Conditions for the proper functioning of the service	52.4 ± 19.7	47.1 ± 18.5	54.6 ± 16.4	55.8 ± 20.7	0.246
Factor 4 - Organization and sustainability of nursing practice	60.0 ± 13.1^a^	60.7 ± 19.4^a^	71.7 ± 15.5^b^	68.5 ± 18.5^b^	0.043
Factor 5 - Institutional policy for professional qualification	60.6 ± 16.2	58.2 ± 24.2	69.4 ± 20.4	67.2 ± 20.1	0.123
Factor 6 - Quality and safety of nursing care	66.9 ± 17.9^ab^	59.5 ± 21.3^a^	70.7 ± 22.2^ab^	71.4 ± 17.5^b^	0.038
Process	71.6 ± 11.0^a^	72.1 ± 12.2^a^	79.8 ± 10.9^b^	78.0 ± 12.6^b^	0.025
Factor 1 - Collaboration and teamwork	71.2 ± 15.1	72.9 ± 14.0	79.5 ± 10.9	74.9 ± 14.2	0.227
Factor 2 - Strategies for quality assurance of care	67.7 ± 12.1^a^	67.3 ± 16.0^a^	77.9 ± 15.0^ab^	78.1 ± 15.7^b^	0.002
Factor 3 - Autonomous practices in professional practice	73.6 ± 10.4	74.2 ± 13.7	76.2 ± 14.5	73.6 ± 16.0	0.904
Factor 4 - Theoretical and legal subsidies for professional practice	79.0 ± 12.5	80.6 ± 17.7	86.7 ± 16.0	84.0 ± 15.1	0.335
Factor 5 - Interdependence in professional practice	66.4 ± 17.2^a^	65.5 ± 16.6^a^	75.4 ± 19.1^ab^	78.2 ± 17.4^b^	0.003
Results	60.8 ± 16.0^ab^	55.7 ± 20.3^a^	69.0 ± 15.5^b^	68.0 ± 16.6^b^	0.005
Factor 1 - Systematic evaluation of nursing care and indicators	63.0 ± 19.0^a^	58.1 ± 19.7^a^	76.0 ± 14.9^b^	71.7 ± 18.4^b^	0.001
Factor 2 - Systematic evaluation of nurses’ performance and supervision	57.1 ± 14.9	53.4 ± 24.5	60.5 ± 21.1	64.2 ± 18.2	0.070

* One-way Analysis of Variance (ANOVA); ^a,b^ Equal letters do not differ by Tukey’s test at a 5% significance level.

## Discussion

In Brazil, there is a majority of young professionals working in the FHS. This is due to changes in the undergraduate curriculum, which have increased actions and new educational programs for better professional qualification (12, 13) and in this research, the profile of working professionals is not dissociated from this data.

When it comes to evaluating professional practice environments, it can be seen that in the Structure dimension, there is a preponderance of positive evaluations for people management and nurse leadership. The structure refers to organizational factors, aspects related to training, innovation, and research, associated with quality and safety of care, involves the management of people and material resources and others related to the organization and sustainability of nursing practice, and factors related to leadership management in the service (14).

Multidisciplinary teamwork and effective management are essential to improving the quality of health care, making it more coordinated and patient-centered. Positive evaluations of the environment and recognition increase satisfaction, especially among technicians and assistants. Nurse leadership, collaboration between teams, and strategies to ensure quality positively influence structure and processes, although licensed nurses report lower satisfaction in these areas (15, 16).

Health management involves coordinating and directing health systems at different levels of government, whether at municipal, state or national level, requiring a robust set of skills to efficiently manage the human resources involved (17). At the same time, leadership in the healthcare context is often associated with the ability to set clear objectives and guide multidisciplinary teams to achieve them, creating an environment that fosters employee engagement and job satisfaction (18).

This finding can be extrapolated to other contexts, indicating that investing in training nursing leaders contributes to the efficient organization of health services, especially in primary care, where team integration is fundamental to the success of care actions.

Both contribute to the organization and coordination of the nursing team, ensuring efficient quality in the work process, bringing effective results for the quality of care provided to the community, which in turn requires professionals to have knowledge and skills not only in care, but also in management, which are the responsibility of nurses alone (19).

Evaluating care services is one of the regulations governing nursing practice, according to Law No. 7,498 (1986). In addition, professional practice environments that are favorable to working conditions bring professional satisfaction and consequently better work performance (7).

Linked to this factor, nursing technicians and assistants had more positive evaluations in the organization and sustainability of nursing practice compared to nurses. Although the Brazilian reality is unique in terms of the social division of labor, the data indicate the importance of reducing professional turnover and investing in planning nursing activities.

Potentially, nursing technicians and assistants rated the methodologies for organizing nursing care positively, which according to other authors indicates that the environment allows them to better mediate the workload, respond to patients’ needs, and reduce the risk of adverse events (20).

It should be noted that regardless of personal or professional characteristics, the entire nursing team can contribute to sustainable practices and the organization of health services (21) and the result of these factors (physical environment, material resources, and personnel) comes from the perception of the professionals interviewed (22), as they are facilitators of the nursing work process and are more active in practical care activities aimed at caring for people, families and the community (23).

It is also noteworthy that the nursing assistants had a significantly higher score for the quality and safety of nursing care. This factor assessed sizing and whether the nursing work methodology adopted promotes quality of care and guarantees safe practices. The result shows that there is a higher prevalence of auxiliary professionals in the PHC when compared to the number of nurses in the sector, which may have contributed to this outcome (24).

The sizing of nursing staff is based on COFEN Resolution 543/2017, which establishes parameters for sizing the number of professionals needed to work in a given health service (20). However, it presents limits for sizing in PHC. A study on the subject reports on the challenges faced within the PHC to guarantee the diversity of activities undertaken by nursing with the number of staff available, with warnings about recording information, monitoring data, guaranteeing spaces for team meetings, and shows the need for more research on the subject (25).

In the Process dimension, the most positively evaluated factors were collaboration and teamwork, strategies for quality assurance of care and Interdependence in professional practice.

Regarding collaboration and teamwork, the study highlights that they involve: autonomy in decision-making, updating care plans, effective communication between team members ensuring adequate planning, good working relationships between different team members, electronic document management, working relationships between doctors and nursing professionals, understanding and valuing their respective roles and responsibilities and care based on health promotion (10).

In line with the result obtained, collaboration in teamwork is potentially the result of an organized place, through dialogue and team meetings are scheduled monthly (26), in this regard, the nurse manager’s performance influences the team’s work (27) and he must act with theoretical and practical support that regulates professional practice (28).

Due to the division of the work process, nurses need to act in a more judicious, innovative way and focus on management, quality, care and the therapeutic process, assuming responsibility for the managerial activities of health services and the entire nursing team (29). On the other hand, nursing technicians and assistants are active in all sectors of PHCs in care activities, closer to the practices and direct contact with the user (30).

Regarding the evaluation of Interdependence in professional practice, researchers analyze whether the practice of nursing professionals is fundamentally focused on managing signs and symptoms of the disease, the attention of professionals in responding to prescriptions from other professionals in a clear appreciation of interdependence and whether they have the perception that with the implementation of interdependent interventions, the work is done (10). In this last item, the valuing of autonomy in professional practice is noticeable when care models are adopted that are centered on people rather than pathologies, which contributes to stewardship in professional practice (31).

Outcome dimension shows desirable or undesirable changes and is considered the final component of the evaluation of the institution, care, patients, and professionals (9). A study in a hospital setting found a relationship between gender and the Systematic evaluation of nursing care and indicators, with higher scores among women, which may be associated with the ease with which women perform care in a more naturalized way compared to men (32).

Quality indicators and systematic assessment are essential to monitor and implement improvements, ensuring safe and efficient care. Well-managed environments promote safety and a healthy workplace, directly impacting the care provided (33). The value of this monitoring was more evident among female professionals, revealing the influence of individual perceptions on perceived quality. Since the beginning of its precursors, nursing has been carried out by women, contributing to the feminization of health (23). However, it is worth noting that 5% of the 159 interviewees belong to the male category, despite being a minority there has been an increase in the number of professionals of this gender in recent years at the FHS (34).

Effectively, the Results show the outcome and changes that occurred during the processes, which can be characterized by the satisfaction or dissatisfaction of users and professionals, complications during care and knowledge acquired by them during the care process (35). In thi6s context, quality indicators come as a complement to the dimensions, as they seek to identify opportunities to improve health, they are tools used to guide planning policies, program development, and health financing, the results obtained are essential to evaluate the performance of PHC service actions (36).

In this sense, the funding model in force in Brazil, Previne Brasil, reinforces the use of these indicators, which establishes the standard to be monitored, strengthening the care provided by the nursing team, as they are one of the main coordinators of care (37). However, there is a need to expand specific indicators for monitoring results beyond programmatic demands, with a return to health promotion actions in the territory, essential in the FHS model, in which nursing plays an important role (38).

It is worth noting that the overall evaluation of health services shows satisfactory results (39) and is fundamental to a culture of safety.

Nursing technicians and assistants also scored significantly higher on the Systematic evaluation of nursing care and indicators, and on the global Outcome scale, compared to care nurses. Professionals with graduate degrees, on the other hand, had significantly lower total scores compared to those with higher education.

Regarding the level of education, no studies were identified that discuss the subject, however, it is worth mentioning that this factor may be associated with the critical sense that professionals develop during their training, that is, the higher the level of education, the greater the critical sense and their negative perception of care, as they are more demanding of quality indicators, since these professionals seek improvements and innovations for health services.

A study in the Portuguese hospital setting, without the presence of nursing technicians and assistants, highlights the need to invest in a sustained training path oriented toward work methods aimed at quality of care (40), aspects that are still challenging in the context of Brazilian nursing in PHC, where there is a unique care model, with a diversity of professional categories.

Effective leadership in nursing is fundamental to facing the challenges presented in primary care environments, especially in health systems with complex care models such as the Brazilian one. Trained leaders can positively influence the work dynamic and interaction between the various professional categories, promoting a culture of collaboration and mutual respect. This leadership should be characterized by the ability to make evidence-based decisions and the ability to motivate and engage the team in the continuous pursuit of excellence in care, thus ensuring a more effective response to patients’ needs. Therefore, investment in leadership development in nursing is as crucial as technical training, as it is the key to transforming the quality of healthcare at all levels of care (2).

The findings of this study highlight the importance of investing in research into the evaluation of professional nursing practice environments in primary care settings, which is crucial to increasing the quality of access to health services.

## Conclusion

The findings indicate an environment favorable to the quality of nursing care, with the most positive evaluation among nursing technicians and assistants. People management and nurse leadership, collaboration and teamwork, strategies for quality assurance of care, and Interdependence in professional practice were the aspects that most raised the evaluation of Structure and Process, noting that graduate professionals had lower scores in all three dimensions.

Systematic evaluation of nursing care and indicators also proved to be the most valued factor in the Outcome, and was markedly influenced by the female gender.

In regions where PHC is being strengthened, such as in many developing countries, the lessons learned from this study can guide the implementation of evidence-based practices and the continuous improvement of work environments. Investing in the continuous training of nursing professionals is also an aspect that appears to be relevant, especially in places where there is a shortage of qualified professionals or in settings with high turnover.

We can point out as limitations to the study the variability in the conceptualization of “Professional Practice Environment,” which can vary depending on local standards and guidelines, and this can lead to different interpretations by the study participants, resulting in heterogeneous responses. On the other hand, a diversity of organizational structures can be identified as a limitation. The practices and working conditions of nursing professionals can vary widely between different PHC units due to variations in local policies, infrastructure and management. These structural differences can make comparison between units difficult and interfere with the identification of factors that influence professional practice. However, it is hoped that the study will provide information that will enable managers/researchers to analyze the quality of professional nursing practice environments and, based on this, the care provided, in addition to making it possible to monitor and improve these nursing practice environments, identifying aspects that need to be improved.

## Data Availability

The original contributions presented in the study are included in the article/supplementary material, further inquiries can be directed to the corresponding author.
